# Fruit quality assessment based on mineral elements and juice properties in nine citrus cultivars

**DOI:** 10.3389/fpls.2023.1280495

**Published:** 2023-11-30

**Authors:** Yiling Jiao, Shuozhen Zhang, Haitao Jin, Yuwen Wang, Yamin Jia, Hua Zhang, Yuying Jiang, Wenqiang Liao, Li-Song Chen, Jiuxin Guo

**Affiliations:** ^1^ Fujian Provincial Key Laboratory of Soil Environmental Health and Regulation/International Magnesium Institute, College of Resources and Environment, Fujian Agriculture and Forestry University, Fuzhou, China; ^2^ Forestry Science and Technology Test Center of Fujian Province, Zhangzhou, China; ^3^ College of Forestry, Guangxi University, Nanning, China; ^4^ Station of Cropland Construction and Soil and Fertilizer of Fujian Province, Fuzhou, China

**Keywords:** citrus fruits, mineral characteristics, juice properties, fruit quality assessment, fruit quality index

## Abstract

**Introduction:**

Citrus fruit is considered a superfood due to its multiple nutritional functions and health benefits. Quantitative analysis of the numerous quality characteristics of citrus fruit is required to promote its sustainable production and industrial utilization. However, little information is available on the comprehensive quality assessment of various fruit quality indicators in different citrus cultivars.

**Methods:**

A total of nine different fresh citrus fruits containing seeds were collected as the experimental materials. The objectives of this study were: (i) to determine the morphological and juice properties of citrus fruits, (ii) to measure the mineral elements in the peel, pulp, and seeds, and (iii) to evaluate the fruit quality index (FQI) using the integrated quality index (IQI) and the Nemoro quality index (NQI) methods.

**Results:**

There were significant differences in fruit quality characteristics, including morphological, mineral, and juice quality, among the investigated citrus cultivars. The proportion of pulp biomass was the highest, followed by that of peel and seeds. N and Cu had the highest and lowest concentrations, respectively, among the measured elements across all citrus fruits, and the amounts of N, P, Mg, Cu, and Zn in seeds, K and Al in pulp, and Ca, Fe, and Mn in peel were the highest, dramatically affecting the accumulation of minerals in the whole fruit and their distribution in various fruit parts. Additionally, Ningmeng fruits had the highest vitamin C and titratable acidity (TA) but the lowest total soluble solids (TSS) and total phenolic (TP) contents, resulting in the lowest TSS/TA and pH values. In contrast, Jinju fruits had the highest TSS and TP contents. Based on the mineral element and juice quality parameters, principal component analysis showed that the citrus fruits were well separated into four groups, and the dendrogram also showed four clusters with different distances. The FQI range based on the IQI method (FQI_IQI_) and NQI method (FQI_NQI_) was 0.382-0.590 and 0.106-0.245, respectively, and a positive relationship between FQI_IQI_ and FQI_NQI_ was observed.

**Conclusion:**

Our results highlight the great differences in mineral and juice characteristics among fruit parts, which mediated fruit quality. The strategy of fruit quality assessment using the FQI can be expanded for targeted utilization in the citrus industry.

## Highlights

1. Nine citrus fruit cultivars with seeds were selected to comprehensively assess fruit quality.
2. Fruit quality indicators, including morphological, mineral, and juice properties, were determined.
3. Two quantitative methods of the IQI and NQI were employed to conduct the fruit quality assessment.
4. Differences in the fruit quality index were calculated using mineral element and juice quality parameters.

## 1 Introduction

Sustainable crop production and waste utilization are two critical challenges to coordinating food security and environmental protection for a growing global population. Due to their high nutritional value, favorable economic return, and strong environmental adaptability, citrus fruits are one of the most widespread fruit crops and the most commonly consumed fruits (Liu et al., 2012; Khalil et al., 2022). In 2020, the citrus planting area and fruit yield were approximately 10.1×10^6^ ha and 15.8×10^7^ t worldwide (FAOSTAT, 2020), respectively. China is the world’s largest citrus producer, with the planting area and fruit yield reaching 3.0×10^6^ ha and 4.5×10^7^ t and representing 30% and 28% of the worldwide citrus production, respectively (FAOSTAT, 2020). In China, citrus planting is mainly distributed in the hilly and mountainous regions of the southern Yangtze River mainly under rain-fed agricultural ecosystems (Xian et al., 2022; Zhao et al., 2023). In addition, there are rich germplasm resources of citrus fruits, which often include orange, mandarin, lemon, grapefruit, pomelo, and others, and different citrus varieties are planted in various production areas due to the stronger adaptability of planting and grafting, resulting in differences in fruit quality characteristics (Zhou et al., 2018; Guo et al., 2023). Additionally, citrus fruits have diverse flavors and aromas and are considered a superfood for being rich in multiple minerals and bioactive substances, such as K, Ca, Mg, carotenoids, flavonoids, phenolics, and vitamins (Topuz et al., 2005; Barros et al., 2012; Juhaimi et al., 2018; Kumar et al., 2022). Furthermore, inorganic elements in citrus fruits have various biochemical functions in living organisms, and bioactive compounds play an essential role in nutrition and biological processes, including anti-oxidant, anti-inflammatory, anti-aging, and anti-cancer activities for improving human health (Lagha-Benamrouche and Madani, 2013; Escudero-López et al., 2018; Guo et al., 2020; Zayed et al., 2021). Therefore, citrus fruits have been widely applied in the food, cosmetics, and medical industries.

Most citrus fruits contain three parts—peel, pulp, and seeds—and are divided into two main types, seeded fruits and seedless fruits. Citrus fruits are mainly used in the food industry as fresh fruits or to produce products such as juice-based drinks. Approximately 26% of citrus fruits are used for making juice, and one-third of all citrus fruits are processed into juice, candies, jams, and puree, resulting in a significant amount of waste citrus peel and seed components being produced after industrial processing (López et al., 2010; Silva et al., 2017; Khalil et al., 2022). The nutritional and biochemical characteristics of citrus residues, especially the peel, which accounts for a large biomass proportion of the whole fruit (40%), and their high value-added reuse have received increasing attention (Ricci et al., 2019; Di Rauso Simeone et al., 2020; Guo et al., 2020; Liu et al., 2021; Nateghpour et al., 2021). In addition, although seedlessness is a desirable characteristic in citrus crops for fresh consumption and the processing industry, citrus seeds have essential and unique biological functions and value as a material for germplasm resource innovation and genetic improvement and are also rich in mineral and bioactive substances for industrial utilization (Juhaimi et al., 2018; Neves et al., 2018; Zayed et al., 2021). With the increasing consumer and processing industry demand for higher quality fruit, precise production and waste management have steadily increased (Omran et al., 2021; Khalil et al., 2022). Thus, evaluating the fruit quality of different citrus cultivars and various fruit parts using numerous fruit quality indicators is very important and will help to develop effective strategies for fruit quality assessment to enhance citrus production and industrialization.

Assessing the composition and content of nutrients and bioactives of citrus fruits is not easy, which is influenced by many factors, including citrus varieties, fruit parts, fruit maturity, soil type, climate, and even human practices, such as cultivar and fertilization (Lu et al., 2021). In order to provide a scientific basis for farmers and consumers to plant and choose citrus varieties with excellent nutritional quality, a comprehensive evaluation and comparison of the properties of citrus fruits are necessary. Previous studies have confirmed that the integrated quality index (IQI) method is the most commonly used, widely accepted, and efficient method to assess soil quality (Guo et al., 2019; Guo et al., 2022), water quality (Mukate et al., 2019), and plant stress tolerance (Sun et al., 2018) under various conditions using multiple quality indicators. This is mainly due to its quantitative flexibility and high operability in the assessment process given the diversity of measured indicators or parameters. The IQI model was initially developed from the soil quality index assessment, in which high IQI values represent high soil quality and soil function (Liebig et al., 2001). It has the advantage of integrating systematic complexity effects to evaluate the relationships between soil quality indicators and productivity (Guo et al., 2019; Guo et al., 2022). Additionally, the Nemoro quality index (NQI) is a quantitative model that has been applied and compared with the IQI model in soil quality assessment (Qi et al., 2009). In citrus fruits, common indicators of fruit quality include juice quality parameters, such as total soluble solids (TSS), titratable acidity (TA), and TSS/TA; additionally, the mineral elements, including N, P, and K, are regarded as the critical factors of fruit quality (Khalid et al., 2012; Zhou et al., 2018). However, little is known about the selection of the optimal indicator for the evaluation of fruit quality in different citrus fruits, and even less information is available on the comprehensive assessment of the diversified fruit quality indicators to obtain a quantized value with the fruit quality index (FQI) based on IQI and NQI methods, especially for the contribution from different citrus fruit parts.

Accordingly, the aim of this study was to objectively evaluate the FQI of citrus fruits using the IQI method in combination with the NQI method, primarily by investigating the characteristics of mineral concentration, accumulation, and distribution in various citrus fruit parts, including the peel, pulp, and seeds in nine citrus fruits. Additionally, the fruit morphological and juice quality properties were determined. These results will provide valuable insights into potential strategies for sustainable production and waste utilization of citrus fruits.

## 2 Materials and methods

### 2.1 Sample collection and preparation

A total of nine different fresh citrus fruit cultivars containing seeds were studied as the experimental materials, including *Citrus japonica* (cv. Jinju), *Citrus limon* (cv. Ningmeng I and Ningmeng II), *Citrus reticulata* (cv. Facaigan, Lugan, Maogugan, Miju, and Wogan), and *Citrus sinensis* (cv. Xiacheng), belonging to the genus *Citrus* L. in the family Rutaceae. In February, all fruit samples were collected from the local fruit supermarket of Fuzhou City, which is located in Fujian Province, one of the main citrus-producing areas in China. All mature fruit samples were carefully selected to ensure consistency in size, shape, color, and appearance. The citrus fruits were manually cleaned, washed twice with tap water and deionized water, and dried with paper towels. For sampling, clean citrus fruits were divided into three parts, including peel, pulp, and seeds, and then the pulp was squeezed into juice and immediately placed in liquid N and stored at -80°C until analysis. All parameters were measured in quadruplicate.

### 2.2 Fruit morphological measurements

Fruit morphological characteristics, including fruit length (L) and diameter (D), were measured using a digital caliper (0.1 mm). Then, the fruit shape index (FSI), geometric mean diameter (GMD), sphericity (Ø), and surface area (SA) were calculated by using the following equations (Topuz et al., 2005):


(Eq. 1) FSI=L/D

(Eq. 2) $$\text{GMD}={\left(\text{L}\times {\text{D}}^{2}\right)}^{1/3}$$

(Eq. 3) Ø=GMD/L

(Eq. 4) $$\text{SA}=\text{Π}\times {\text{GMD}}^{2}$$

Additionally, the fruit fresh and dry weights were determined, in which the dry weights of the peel, pulp, and seeds were measured after the samples were oven-dried at 105°C for 30 min and then at 70°C to a constant weight. Finally, the biomass distribution was calculated as the percentage of each fruit part’s biomass to the whole fruit biomass.

### 2.3 Mineral element measurements

To determine the concentrations of minerals in the fruits, including N, P, K, Ca, Mg, Fe, Mn, Cu, Zn, and Al, the dried and ground peel, pulp, and seed samples were wet-digested by the H_2_SO_4_-H_2_O_2_ method for N determination and by the HNO_3_-HClO_4_ method (4:2 v) for other elements (Khalid et al., 2012; Chen et al., 2020; Xu et al., 2022). When samples were completely digested and a clear solution was obtained, the N concentration was measured by an AA3 digital colorimeter (Bran + Luebbe, Hamburg, Germany), and the other mineral elements were measured by an Optima 7300 DV inductively coupled plasma optical emission spectrometer (ICP-OES, PerkinElmer, MA, USA). Additionally, the standard citrus leaf sample (GBW10020, Institute of Geophysical and Geochemical Exploration, Chinese Academy of Geological Sciences) was applied as a reference material to correct the mineral measurements. Nutrient accumulation in the whole fruit was calculated as the sum of each fruit part’s nutrient concentration × dry weight. The nutrient distribution in different fruit parts was calculated as the nutrient accumulation in each fruit part to that of the whole fruit.

### 2.4 Juice quality measurements

Fresh juice samples were used to determine some physico-biochemical characteristics of fruit quality, including total soluble solids (TSS), titratable acidity (TA), TSS/TA, pH, vitamin C (Vc), and total phenolics (Khalid et al., 2012; Gullo et al., 2020). Briefly, the TSS content was measured using a handheld digital refractometer (PR-1; Atago, Tokyo, Japan); the TA content was titrated with 0.1 mol L^-1^ NaOH, and then the TSS/TA was calculated. The juice pH was determined using a pH meter (Orion Star A215, Thermo Scientific, USA). The Vc content was measured using a standard method based on the reduction of 2,6-dichlorophenolindophenol (DIP) dye by ascorbic acid. The total phenol content was measured using the Folin-Ciocalteu method.

### 2.5 Fruit quality evaluation

According to previously described methods (Liebig et al., 2001; Qi et al., 2009; Sun et al., 2018; Guo et al., 2019; Guo et al., 2022), the IQI and NQI were employed to assess citrus fruit quality. There was a four-step process involving indicator selection, weight assignment, scoring, and IQI or NQI calculation. In the present study, 10 mineral elements in the peel, pulp, and seeds, respectively, and 6 juice quality properties were adopted as fruit quality indicators, and the indicator weight assignment was calculated by principal component analysis (PCA). Moreover, the standard scoring function (SSF) and IQI or NQI were applied to calculate the indicator scoring and fruit quality index (FQI), respectively, as follows:


(Eq. 5) Si=0.1+0.9×(i2−i)/(i2 −i1)

(Eq. 6) Si=0.1+0.9×(i−i1)/(i2 −i1)

(Eq. 7) $${\text{FQI}}_{\text{IQI}}=\sum_{i=0}^{n}\text{W}i\times \text{S}i$$

(Eq. 8) $${\text{FQI}}_{\text{NQI}}=\sqrt{\frac{\text{S}ave^{2}+\text{S}min^{2}}{2}\times \frac{n-1}{n}}$$

where *i* is the value of a fruit quality indicator measurement, *i1* and *i2* represent the minimum value and maximum value among different citrus fruit species, S*i* is the indicator score, W*i* is the weight of each fruit quality indicator, S*ave* is the average score of the measured indicators, S*min* is the minimum score of the measured indicators, *n* is the number of indicators, and FQI_IQI_ and FQI_NQI_ represent the FQI based on the IQI and NQI, respectively. However, according to the fruit quality indicator definition of “higher is worse” or “lower is better” for Al concentration and “higher is better” or “lower is worse” for other indicators; the indicator scores of Al concentration and other indicators were calculated using Equations 5 and 6, respectively.

### 2.6 Statistical analysis

All fruit quality indicators were analysed by one-way analysis of variance (ANOVA) using the SAS 9.3 statistical software package (SAS Institute, Cary, NC, USA). Fruit mineral element and juice quality parameters were selected for PCA using SPSS Statistics 21.0 (IBM, Armonk, NY, USA) and plotted using R software. Correlation analysis was conducted using the Pearson product-moment correlation test (two-tailed), while cluster analysis and dendrogram plotting were performed by the nearest distance method. Mean values and correlation coefficients were compared by the least significant difference (LSD) test (*P*< 0.05).

## 3 Results

### 3.1 Fruit morphological properties in different citrus cultivars

There were significant differences in fruit morphological properties among citrus cultivars (**Table 1**). The FSIs were divided into two categories: >1 (including Jinju, Ningmeng I and II) and<1 (including Facaigan, Lugan, Maogugan, Miju, Wogan, and Xiacheng); however, there was a contrasting result for sphericity. The highest fruit length and diameter were found in Ningmeng I and Facaigan, respectively, while Jinju fruits had the lowest fruit length and diameter. In addition, Jinju and Facaigan fruits had the lowest and highest geometric mean diameter and surface area, respectively.

**Table 1:** The physical characteristics of fruits in different citrus cultivars.

Cultivars	Fruit length (mm)	Fruit diameter (mm)	Fruit shape index	Geometric mean diameter (mm)	Sphericity	Surface area (cm^2^)	Fruit fresh weight (g fruit^-1^)	Fruit dry weight (g fruit^-1^)	Peel ratio (%)	Pulp ratio (%)	Seed ratio (%)
Jinju	35.0 ± 1.2 f	30.8 ± 0.7 g	1.14 ± 0.05 c	32.17 ± 0.51 g	0.92 ± 0.03 e	32.51 ± 1.02 g	19.3 ± 0.7 g	3.0 ± 0.1 e	24.2 ± 1.7 g	68.3 ± 2.4 ab	7.5 ± 0.9 a
Ningmeng I	77.7 ± 1.0 a	58.1 ± 2.0 f	1.34 ± 0.05 a	63.97 ± 1.49 cd	0.82 ± 0.02 g	128.56 ± 5.95 cd	130.6 ± 3.0 ef	16.6 ± 0.8 d	48.6 ± 2.7 b	48.5 ± 2.8 e	2.9 ± 0.2 c
Ningmeng II	71.4 ± 0.9 b	58.9 ± 0.9 f	1.21 ± 0.03 b	62.78 ± 0.54 de	0.88 ± 0.01 f	123.76 ± 2.12 de	130.6 ± 3.3 ef	18.0 ± 0.8 cd	46.9 ± 1.0 bc	51.6 ± 1.0 de	1.5 ± 0.2 e
Facaigan	78.9 ± 2.4 a	122.8 ± 1.8 a	0.64 ± 0.03 g	105.91 ± 0.53 a	1.34 ± 0.04 a	352.19 ± 3.54 a	568.4 ± 11.6 a	76.5 ± 3.7 a	60.1 ± 0.8 a	33.8 ± 0.9 f	6.1 ± 0.4 b
Lugan	50.2 ± 1.4 e	71.5 ± 1.1 c	0.70 ± 0.02 f	63.56 ± 0.75 cd	1.27 ± 0.03 b	126.88 ± 2.98 cd	146.3 ± 5.3 d	19.3 ± 0.8 c	28.8 ± 2.4 f	69.1 ± 2.5 a	2.1 ± 0.1 d
Maogugan	52.7 ± 1.2 d	71.7 ± 1.3 c	0.73 ± 0.01 ef	64.71 ± 1.15 c	1.23 ± 0.02 c	131.52 ± 4.67 c	161.6 ± 4.6 c	30.1 ± 0.7 b	29.0 ± 2.7 f	68.4 ± 2.9 ab	2.6 ± 0.2 c
Miju	50.7 ± 1.0 e	68.7 ± 0.4 d	0.74 ± 0.01 ef	62.06 ± 0.66 e	1.22 ± 0.01 c	120.94 ± 2.59 e	133.3 ± 4.3 e	20.1 ± 0.7 c	44.9 ± 1.3 c	54.0 ± 1.3 cd	1.1 ± 0.1 e
Wogan	49.1 ± 1.1 e	65.1 ± 0.8 e	0.75 ± 0.02 de	59.28 ± 0.75 f	1.21 ± 0.02 c	110.35 ± 2.80 f	125.5 ± 3.1 f	20.0 ± 0.6 c	32.9 ± 1.8 e	65.6 ± 1.7 b	1.4 ± 0.1 e
Xiacheng	61.2 ± 1.2 c	77.0 ± 1.1 b	0.79 ± 0.01 d	71.33 ± 1.12 b	1.17 ± 0.01 d	159.79 ± 5.03 b	196.7 ± 2.3 b	29.4 ± 1.0 b	41.5 ± 2.8 d	57.0 ± 2.7 c	1.4 ± 0.1 e

Values represent mean ± standard deviation, and statistical analysis was carried out by ANOVA plus LSD test, and statistical significance (p< 0.05) was indicated with different lowercase letters (a–g).

The lowest and highest fresh fruit weights were found in Jinju and Facaigan, respectively; similar results were observed for dry fruit weights, which ranged from 3.0 to 76.5 g in the investigated varieties. There were significant differences in biomass distribution in different fruit parts, in which the pulp ratio was the highest, followed by the peel and seeds. The exceptions were Facaigan fruits, for which the ratio of peel was higher than that of pulp, and Ningmeng I fruits, for which there were no differences among the parts. The highest proportions of peel, pulp, and seeds were found in Facaigan, Lugan, and Jinju, respectively, and the lowest proportions were found in Jinju, Facaigan, and Miju.

### 3.2 Concentration, accumulation, and distribution of mineral elements in different citrus fruit parts

**Figure 1:**
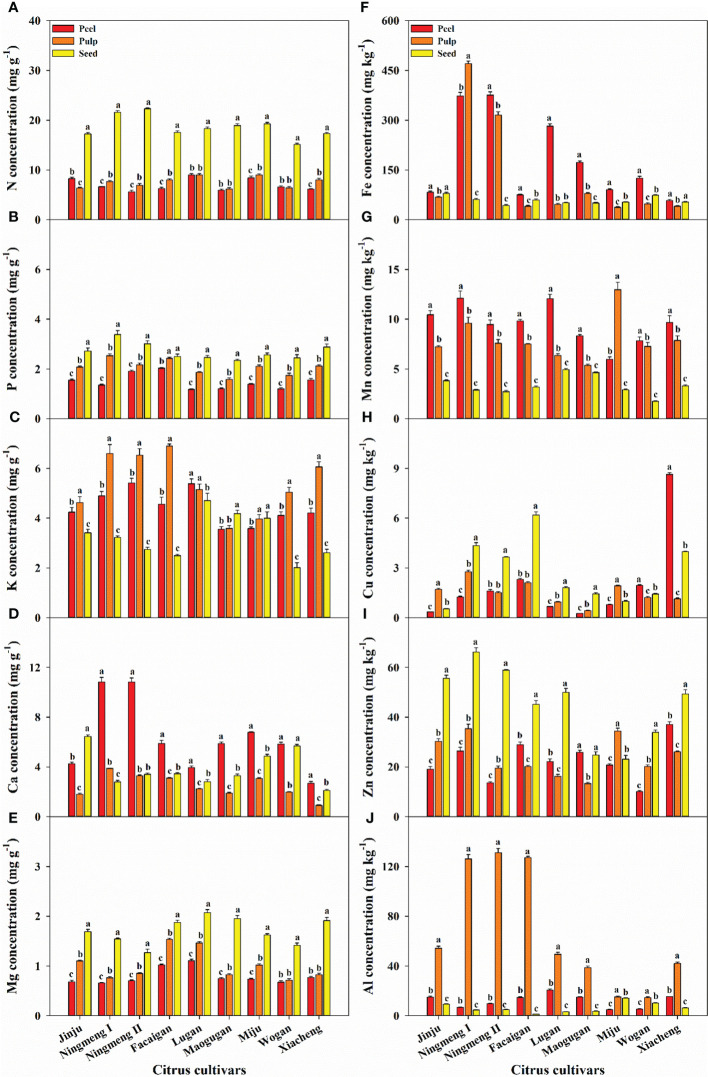
The concentration characteristics of mineral elements of N **(A)**, P **(B)**, K **(C)**, Ca **(D)**, Mg **(E)**, Fe **(F)**, Mn **(G)**, Cu **(H)**, Zn **(I)**, and Al **(J)** in the fruit peel, pulp, and seed organs in different citrus cultivars. The error bars indicate standard deviation; statistical analysis was carried out by ANOVA plus LSD test, and statistical significance (*p*< 0.05) is indicated with different lowercase letters (a–c) in the different fruit organs of the same citrus fruit.

The concentrations of the 10 mineral elements, including macroelements (N, P, K, Ca, and Mg) and microelements (Fe, Mn, Cu, Zn, and Al), are shown in **Figure 1**. N and Cu had the highest and lowest concentrations among the 10 elements, in which the mean N concentrations in the peel, pulp, and seeds were 6.99, 7.51, and 18.63 mg g^-1^ across citrus varieties, respectively (**Figure 1A**), while the mean Cu concentrations were 1.98, 1.53, and 2.71 mg kg^-1^. The concentration of the investigated elements exhibited significant variations among the different fruit parts, with the amounts of N, P, and Mg showing the trend seeds > pulp > peel. K showed the trend pulp > peel > seeds, while Ca was highest in peel followed by seeds and pulp. Among the microelements, the amounts of Fe and Mn showed the trend peel > pulp > seeds, and the amounts of Cu and Zn in seeds were higher than those in peel or pulp, but the amount of Al was highest in pulp followed by peel and seeds.

**Figure 2:**
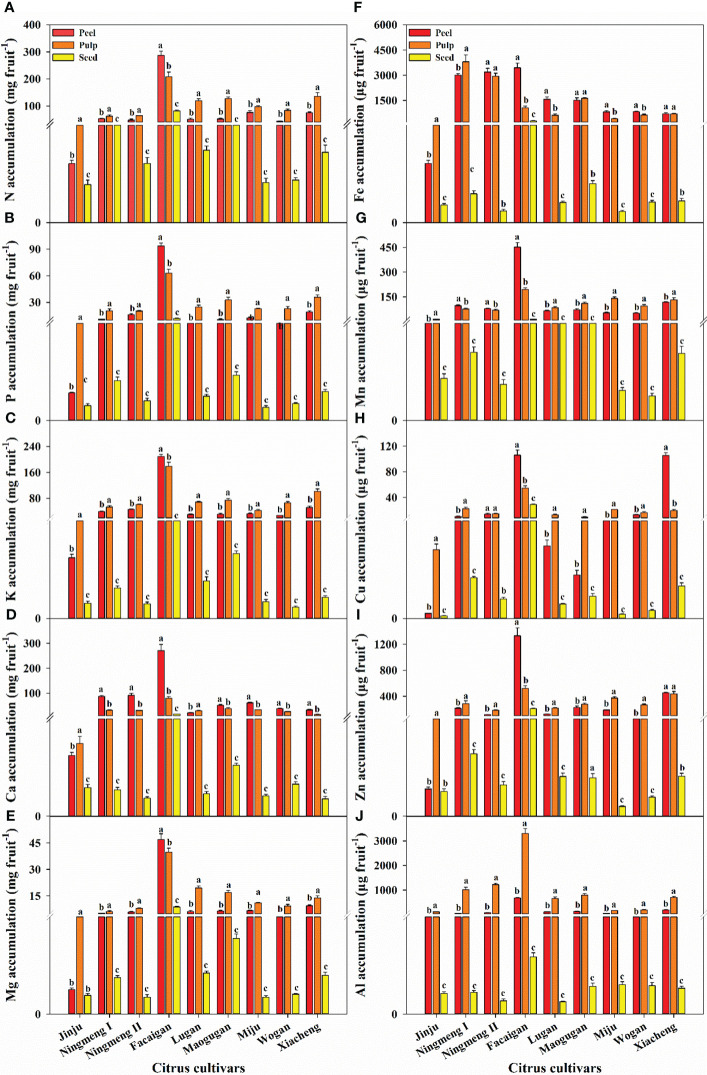
The accumulation characteristics of mineral elements of N **(A)**, P **(B)**, K **(C)**, Ca **(D)**, Mg **(E)**, Fe **(F)**, Mn **(G)**, Cu **(H)**, Zn **(I)**, and Al **(J)** in the fruit peel, pulp, and seed organs in different citrus cultivars. The error bars indicate standard deviation; statistical analysis was carried out by ANOVA plus LSD test, and statistical significance (*p*< 0.05) is indicated with different lowercase letters (a–c) in the different fruit organs of the same citrus fruit.

**Figure 3:**
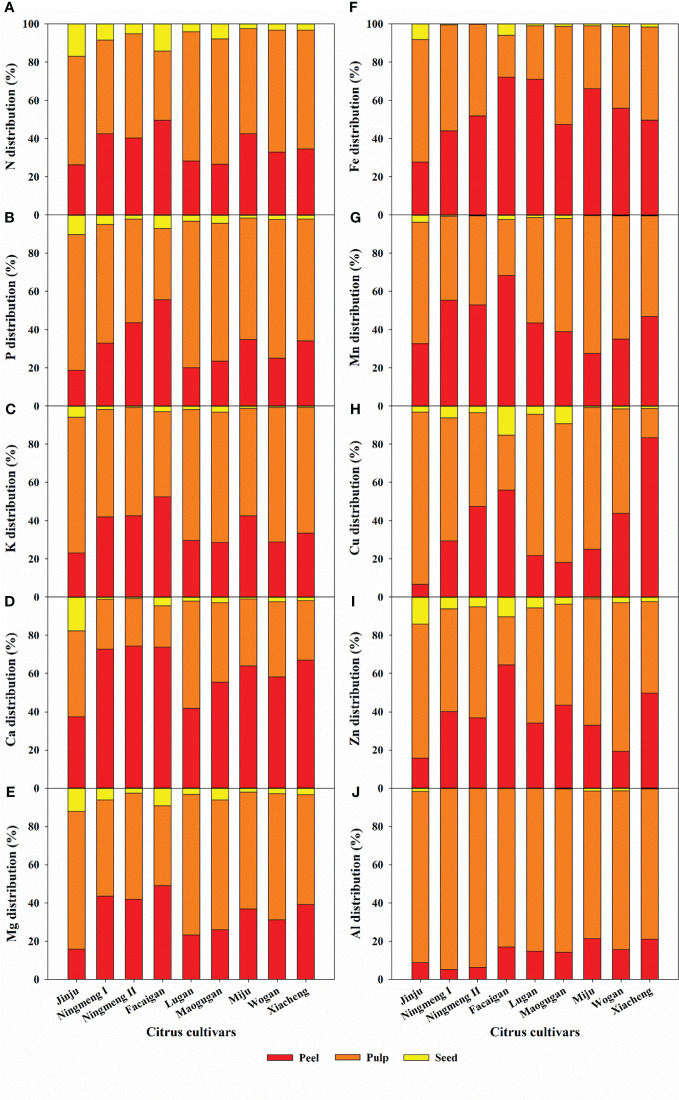
The distribution characteristics of mineral elements of N **(A)**, P **(B)**, K **(C)**, Ca **(D)**, Mg **(E)**, Fe **(F)**, Mn **(G)**, Cu **(H)**, Zn **(I)**, and Al **(J)** in the fruit peel, pulp, and seed organs in different citrus cultivars.

Combined with the dry biomass weight of the corresponding mineral concentration, mineral accumulation in fruit dramatically differed among the different citrus cultivars (**Figure 2**). Except for Ningmeng I fruits having the highest Fe accumulation, the highest accumulation of the other nine elements was observed in Facaigan fruits. Moreover, the distribution of measured mineral elements among the various fruit parts was significantly different in different citrus cultivars (**Figure 3**). As a whole, the proportion of N, P, K, Mg, Mn, Cu, Zn, and Al accumulation in the pulp was always higher than that in the peel across the investigated citrus fruits, in contrast to that of Ca and Fe; however, seeds had the lowest proportion of these measured elements.

### 3.3 Juice quality properties in different citrus fruits

**Figure 4:**
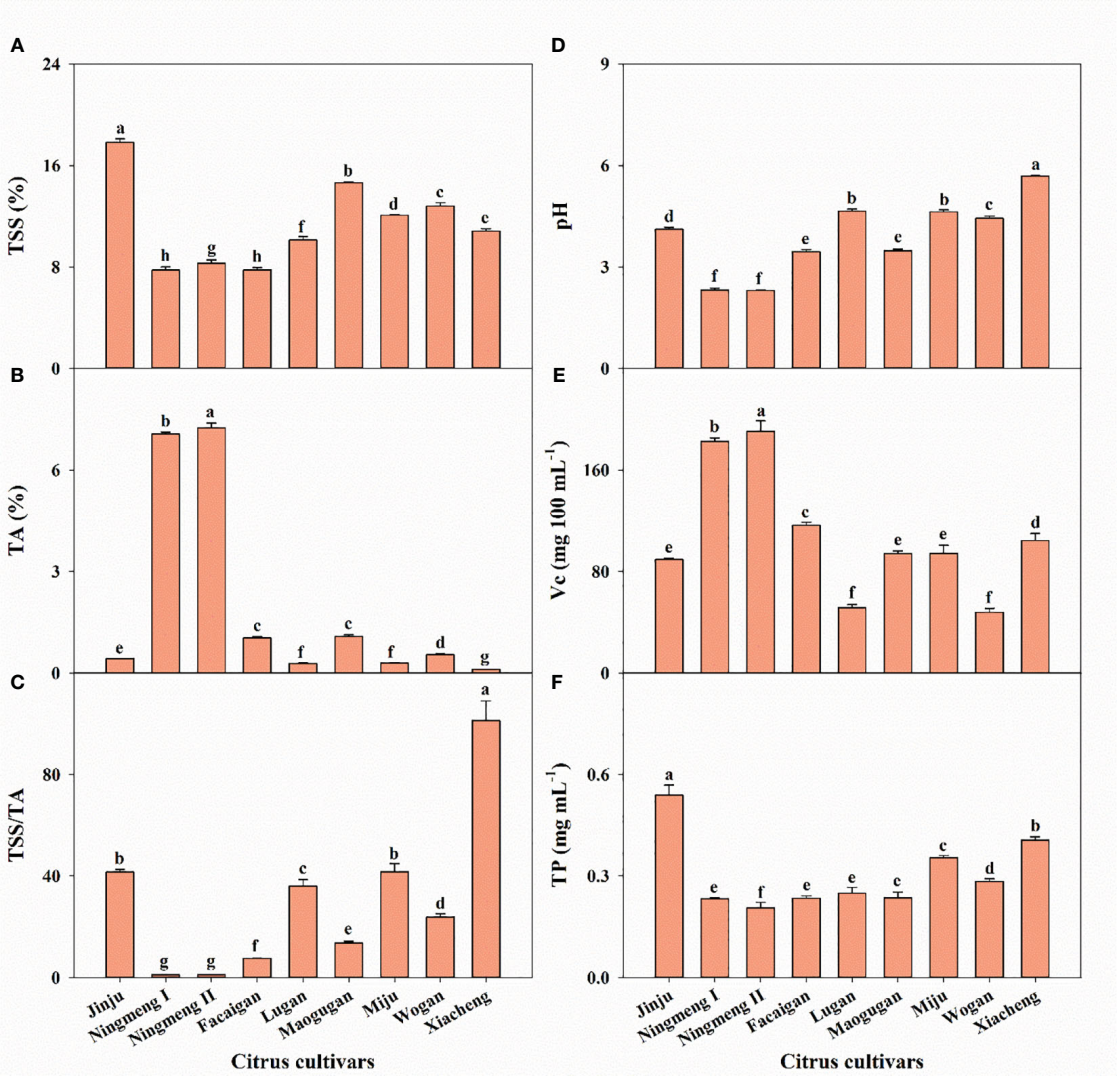
Levels of total soluble solids (TSS) **(A)**, titratable acidity (TA) **(B)**, TSS/TA **(C)**, pH **(D)**, vitamin C (Vc) **(E)**, and total phenolics (TP) **(F)** in the juice of different citrus cultivars. The error bars indicate standard deviation, statistical analysis was carried out by ANOVA plus LSD test, and statistical significance (*p*< 0.05) is indicated with different lowercase letters (a–g) in the different citrus fruits.

There were significant differences in juice quality properties among the different citrus fruits (**Figure 4**). The TSS content was divided into two categories: >10% (including Jinju, Maogugan, Miju, Wogan, Xiacheng, and Lugan) and<10% (including Facaigan and Ningmeng I and II). Similarly, the TA content was divided into two categories: >1% (including Facaigan, Maogugan, and Ningmeng I and II) and<1% (including Jinju, Lugan, Miju, Wogan, and Xiacheng), resulting in the highest and lowest TSS/TA being observed in Xiacheng and Ningmeng, respectively.

The juice pH value and TP content variations were relatively small among the citrus samples, ranging from 2.31 to 5.69 and 0.21 to 0.54 mg mL^-1^, respectively. The lowest and highest Vc contents were found in Wogan and Ningmeng II, respectively, ranging from 47.89 to 190.59 mg 100 mL^-1^ in the investigated fruits. In addition, some positive and negative relationships between juice quality and mineral elements were observed (**Figure S1**).

### 3.4 Fruit quality assessment in different citrus cultivars

**Figure 5:**
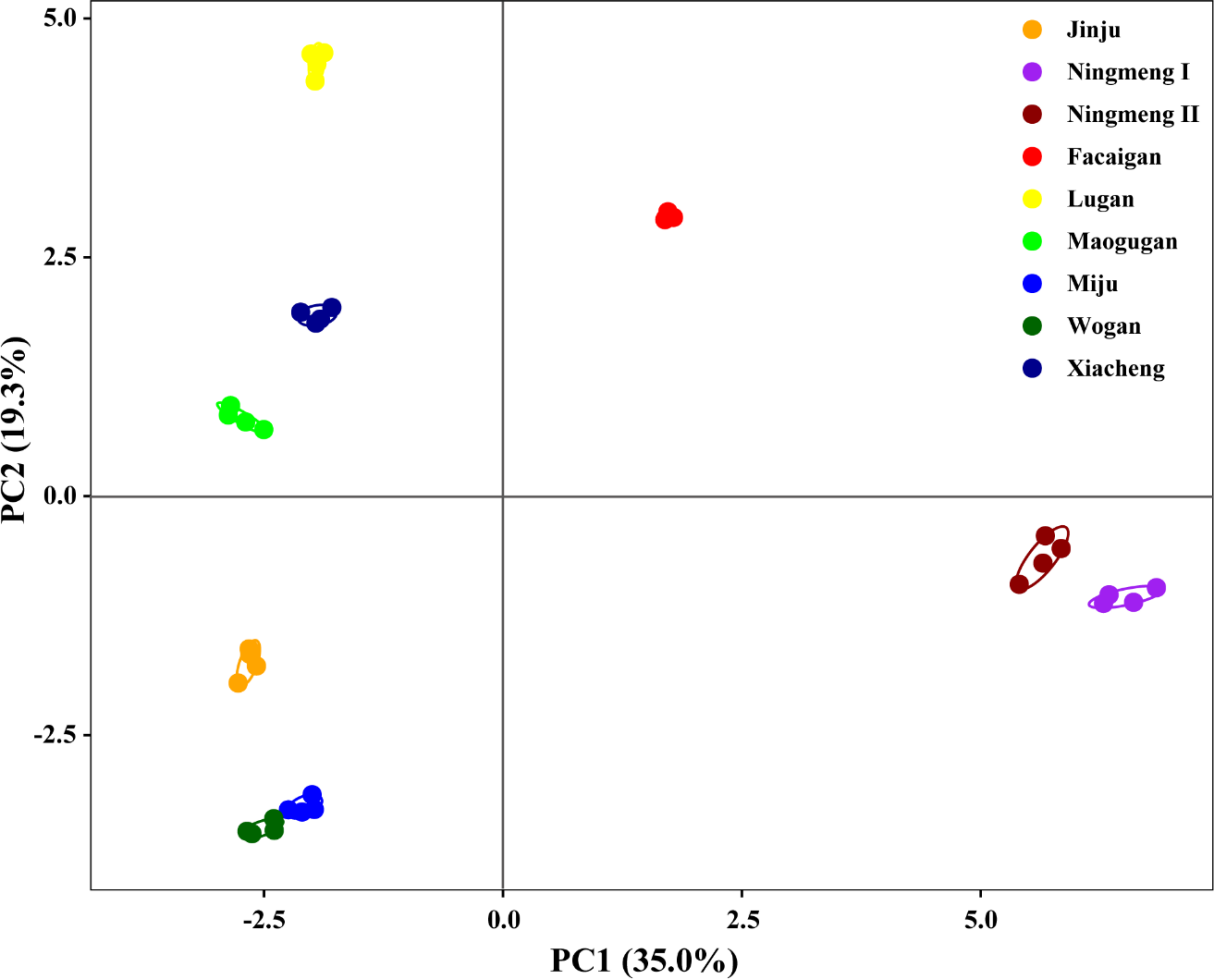
Principal component analysis for fruit peel, pulp, and seed mineral characteristics and juice quality properties in different citrus cultivars.

**Figure 6:**
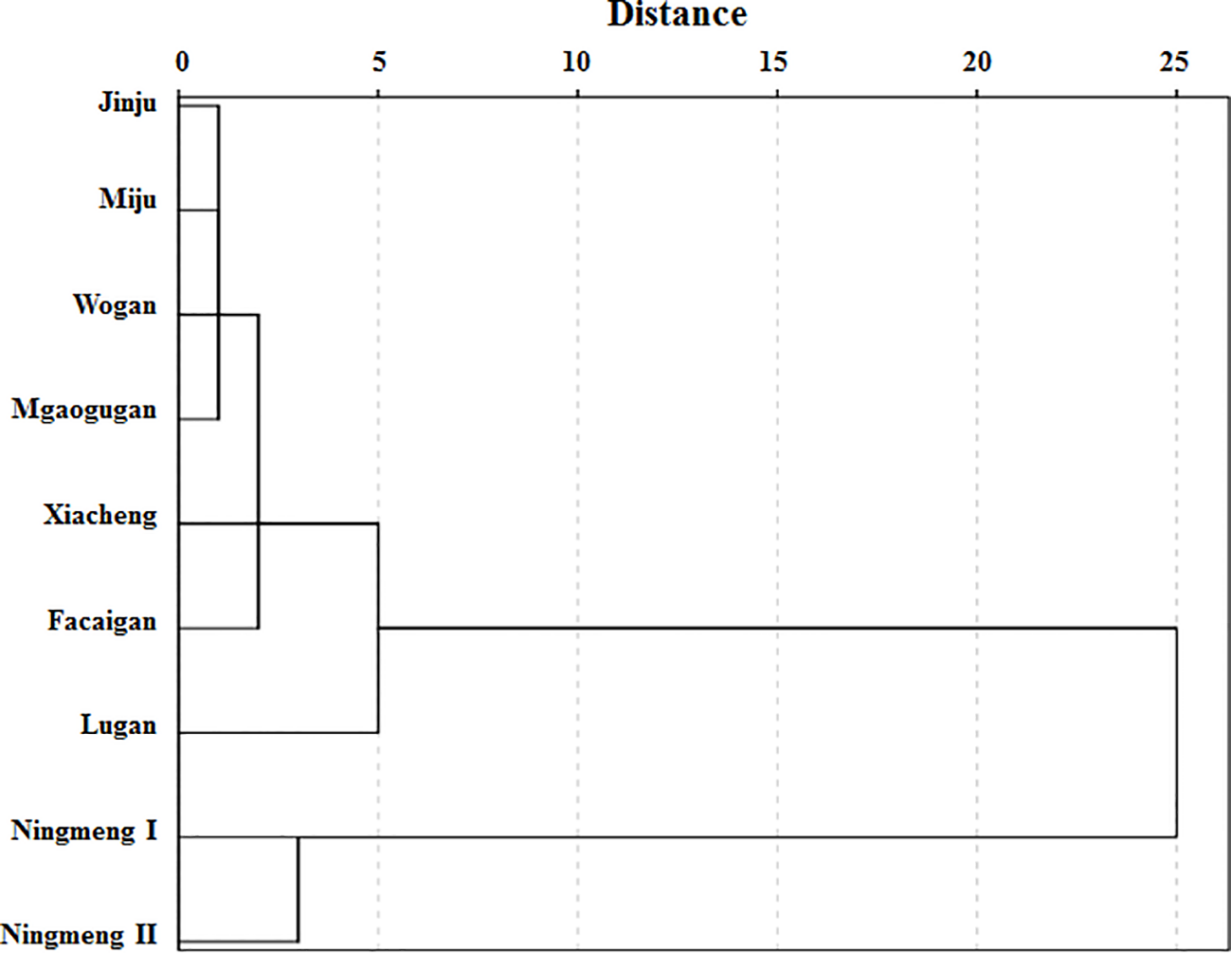
Dendrogram analysis for fruit peel, pulp, and seed mineral characteristics and juice quality properties in different citrus cultivars.

For the investigated citrus cultivars, PCA was conducted with 36 fruit quality indicators, including 10 mineral elements in the peel, pulp, and seeds, respectively, and 6 juice quality properties. Seven main PC groups were obtained, and cumulative values reached 96.1% in the measured indicators (**Table S1**), in which PC1 and PC2 accounted for 35.0% and 19.3% of the total variation, respectively; the different citrus cultivars were well separated and divided into four groups by the PC scores (**Figure 5**). The four main cluster groups observed were formed by Lugan, which had the greatest distance (**Figure 6**), followed by Ningmeng I and II in the third group, Xiacheng and Facaigan in the second group, and Jinju, Miju, Wogan, and Maogugan, which had the shortest distance.

**Figure 7:**
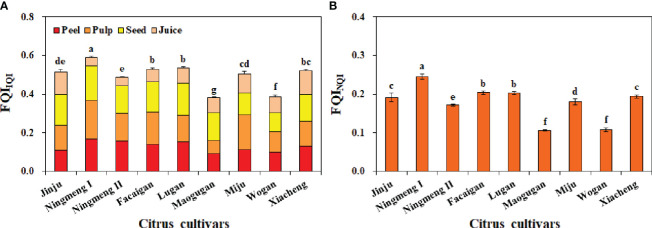
Fruit quality index (FQI) based on the IQI method (FQI_IQI_) **(A)** and NQI method (FQI_NQI_) **(B)** of different citrus cultivars. The error bars indicate standard deviation, statistical analysis was carried out by ANOVA plus LSD test, and statistical significance (*p*< 0.05) is indicated with different lowercase letters (a–g) in the different citrus fruits.

**Figure 8:**
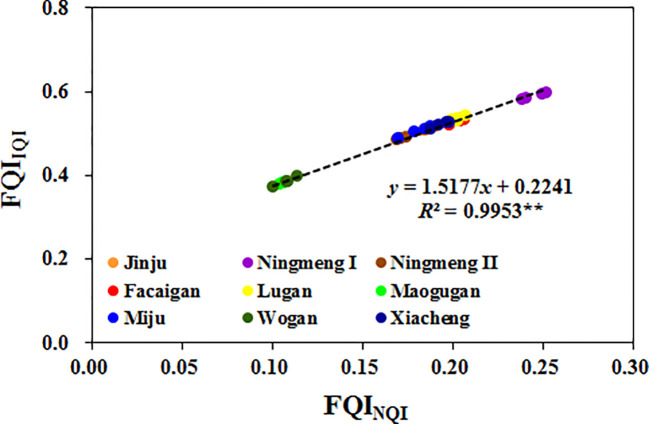
The linear relationship between fruit quality index (FQI) based on the IQI method (FQI_IQI_) and NQI (FQI_NQI_) method of different citrus cultivars.

Similarly, there were significant differences in integrated fruit quality among the different citrus cultivars. The commonalities of fruit quality indicators accounted for 73.38% (Peel P) to 99.64% (Peel Fe) of the proportion of variation (**Table S2**), with these values corresponding to the lowest and highest weights. After fruit quality indicators were scored and weighted, the estimated FQI_IQI_ was 0.516, 0.590, 0.487, 0.530, 0.536, 0.382, 0.505, 0.385, and 0.521 (**Figure 7A**), while the estimated FQI_NQI_ was 0.192, 0.245, 0.172, 0.204, 0.203, 0.106, 0.180, 0.108, and 0.194 (**Figure 7B**) in Jinju, Ningmeng I, Ningmeng II, Facaigan, Lugan, Maogugan, Miju, Wogan, and Xiacheng, respectively. Moreover, there was a positive relationship between FQI_IQI_ and FQI_NQI_ in different citrus fruits (**Figure 8**).

## 4 Discussion

### 4.1 The importance of mineral characteristics to fruit quality in citrus fruits

Fruit quality assessment is a critical strategy to improve fruits and their by-products for deep processing and reutilization in modern agriculture and industry. Mineral elements, both macro- and micro-elements, are the most fundamental nutrition properties for human health and are also regarded as one of the most critical indicators for fruit quality evaluation (Barros et al., 2012; Liu et al., 2012; Chen et al., 2018; Khalil et al., 2022). This is mainly because the inorganic elements in citrus fruits play an essential role in the biological system and have a number of direct and indirect biochemical functions, including providing enough nutrition, regulating enzyme activity, maintaining osmotic pressure and acid-base balance, and mediating metabolic processes (Topuz et al., 2005; Silva et al., 2017; Czech et al., 2020). Therefore, exploring the mineral characteristics, including the concentration, accumulation, and distribution of the given elements, helped reveal the differences among various fruits and parts to understand the mineral nutrition in citrus fruits.

In the present study, the N concentration was the highest among the measured mineral elements in the investigated citrus fruits, and the mean N concentration in seeds was 2.5- and 2.7-fold higher than that in pulp and peel (**Figure 1A**). As a component of protein, a relatively high N concentration means citrus fruits are a rich N source, which indicates that they have an adequate protein content for dietary intake. Moreover, the concentrations of P and Mg in seeds were always the highest, followed by pulp and peel; Ca was the highest in the peel, followed by seeds and pulp; in contrast, K was mainly found in the pulp (**Figures 1B–E**). Higher P and Mg in seeds could provide the functionality required to maintain seed activity and accelerate germination, which helps improve seedling morphogenesis and stress tolerance in plants (White and Veneklaas, 2012; Ceylan et al., 2016; Zhang et al., 2020). Additionally, phytate is the main form of P in grains or seeds, and it cannot be digested by humans and monogastric animals, resulting in reduced P and relative mineral availability due to the strong complexes formed by phytic acid with minerals, such as Zn and Fe (Yamaji et al., 2017). Song et al. (2021) investigated the spatial distribution of phytic acid and mineral availability in four fruit parts, including flavedo, albedo, segment membrane, and juice sac, and found that the inhibitory effect of phytic acid on the availability of Ca, Mg, Fe, Zn, and Mn was less limited in the juice sac than in other parts of five pomelo cultivars. These results suggest that paying more attention to mineral availability in citrus fruits for the human diet is necessary. In addition, K, which regulates the body’s electrolyte and acid-base balance (Czech et al., 2020), was highest in the pulp, indicating that this mineral can be consumed through the diet to meet human needs. Higher Ca in the peel not only helps to resist pests and diseases and prevents fruit cracking but also contributes to the storage and transportation of citrus fruits (Li and Chen, 2017; Silva et al., 2017; Rehman et al., 2018). Moreover, Cu had the lowest concentration, Cu and Zn mainly occurred in seeds, and Fe and Mn mostly occurred in peel and pulp (**Figures 1F–I**). Higher Fe and Zn in the edible parts can protect the body against oxidative stress and improve immune capacity, especially for pregnant women (White and Broadley, 2009). However, Al mainly occurred in pulp (**Figure 1J**), which indicates that Al has a strong transport ability from the plant or other fruit parts to the pulp. However, it is worth noting that heavy metal elements, such as As, Al, Cd, and Pd, are environmental contaminants that can have serious safety risks to plant growth and human health (Czech et al., 2021). Additionally, the uptake properties of rare earth elements, including Ce, La, Sm, Eu, and Sc, in citrus plants were reported (Turra et al., 2019). These results suggested that risk prevention of heavy metals and rare elements in citrus fruits should be performed via monitoring and controlling for a healthy dietary supply.

Our results also showed that the concentrations of K, Ca, Fe, Cu, Zn, and Al have an enormous variation among citrus fruits, in contrast to those of N, P, Mg, and Mn (**Figure 1**), and Ningmeng fruits generally had higher concentrations of K, Ca, Fe, Zn, and Al. However, the highest Cu concentration in the peel was observed in Xiacheng fruits. These results indicated significant differences in mineral elements among citrus fruits and parts, which further implied that the mineral properties of citrus fruits could be jointly regulated by genetics, environment, and management practices (Topuz et al., 2005; Barros et al., 2012; Khalid et al., 2012; Czech et al., 2020). Differences in the concentration of mineral elements in different citrus fruits will directly affect their accumulation and distribution in various fruit parts (**Figures 2**, **3**). The mean accumulation in the whole fruits was 193.57 mg of N, 49.44 mg of P, 127.46 mg of K, 108.27 mg of Ca, and 25.88 mg of Mg, as well as 3049.69 μg, 216.30 μg, 52.15 μg, 632.41 μg, and 1058.13 μg of Fe, Mn, Cu, Zn, and Al, respectively (**Figure 2**). However, the lowest and highest values were observed in Jinju and Facaigan fruits, indicating that mineral accumulation properties were regulated by fruit weight and mineral concentration. Except for Ca (60.51%) and Fe (54.02%), which were mainly distributed in the peel, other mineral elements, including N, P, K, Mg, Mn, Cu, Zn, and Al, were mostly distributed in the pulp, and these proportions ranged from 54.10% for Mn to 85.58% for Al (**Figure 3**). However, the lowest proportion of these measured elements was consistently observed in seeds, ranging from 0.63% for Al to 7.28% for N. These results indicated that the distribution of mineral elements was significantly different in individual fruit parts among citrus fruits, in which the pulp was the main accumulation organ, followed by peel and seeds, further suggesting that both waste citrus peel and seed should be intensively processed for reutilization as high added-value industrial products (López et al., 2010; Ani and Abel, 2018; Czech et al., 2020; Khalil et al., 2022).

### 4.2 The importance of juice characteristics to fruit quality in citrus fruits

Generally, fruit quality parameters, which mostly considered the pulp, especially the content and ratio of juice TSS and TA, differed considerably from the perspective of the market, consumption, and health (Barros et al., 2012; Ani and Abel, 2018; Escudero-López et al., 2018). In this study, our results showed that the highest coefficient variation was 147.25% for the TA indicator of different citrus fruits, followed by TSS/TA (105.44%), sucrose (69.32%), Vc (46.29%), TP (35.96%), and TSS (29.92%), and the lowest was 28.76% for the pH indicator (**Figure 4**). Moreover, Ningmeng fruits had the highest TA and Vc content and the lowest TSS, TSS/TA, pH, TP, and sucrose content; Xiacheng fruits had the highest TSS/TA and pH value; Jinju fruits had the highest TSS and TP content; however, the highest sucrose content was observed in Maogugan fruits. These results indicated large differences in juice quality properties among citrus fruits, which provides a basis for the selective utilization of high-value products. In addition, some juice quality attributes, such as health-promoting nutritional and functional components, including protein, carbohydrates, fat, dietary fibre, ascorbic acid, and flavonoids, as well as antioxidant properties, including the ferric reducing antioxidant power and ABTS cation and DPPH radical scavenging activities, were observed and showed differences among the different citrus fruits (Barros et al., 2012; Wang et al., 2019; Guo et al., 2023). In recent years, citrus fruit residues, including peel and seeds, have been directly used as a substrate to produce animal feed and fertilizer, as well as by biorefinery to produce essential oils, pectin, ethanol, methane, industrial enzymes, and single-cell protein, which have attracted increasing attention from producers, consumers, and processors (López et al., 2010; Liu et al., 2021; Zayed et al., 2021; Khalil et al., 2022). This study quantified the different contributions of peel, pulp, seeds, and juice to the FQI according to the measured fruit quality indicators among citrus fruits (**Figure 7A**). Citrus fruits are widely accepted as a waste-free superfood, suggesting that their multiple fruit quality characteristics are not ignored, including the mineral elements and juice properties in different fruit parts.

The relationship between fruit quality and mineral elements is a scientific problem that researchers and producers are concerned about because it relates to specific cultivation practices, such as fertilization. Li et al. (2015) reported that the content of mineral elements in the leaf was poorly associated with the availability of the corresponding soil elements, and a few positive and negative relationships were observed between fruit quality parameters and soil or leaf elements by a field investigation. Zhou et al. (2018) found that only the Mg concentration was significantly negatively correlated with the sugar component and positively correlated with the organic acid component in pulp compared with other minerals, including N, P, K, Ca, Fe, Mn, Cu, and Zn. In this study, there was a negative relationship between most of the mineral elements and juice TSS, TSS/TA, pH, and TP, and there was a positive relationship between most of the mineral elements and juice TA and Vc (**Figure S1**). Additionally, the juice quality was related to minerals in the peel and seeds across different citrus fruits. Moreover, the fruit morphological properties were significantly different in citrus fruits and even different varieties of the same separate groups (**Table 1**), which are mainly affected by their internal genotypes and external agronomic measures such as fertilization, and the genotype dominates (Guo et al., 2023). However, morphological analyses are still needed not only for identifying and evaluating the genetic diversity and genetic relationship among the citrus groups but also for validating the desired characteristics of varieties, as well as the potential relationships between fruit minerals and morphological parameters observed (Li and Chen, 2017; Zhou et al., 2018; Czech et al., 2020). In summary, these results indicate that fruit morphological characteristics directly affect mineral accumulation in the whole fruit and its distribution in various fruit parts and indirectly affect juice quality, which further regulates fruit quality and consumption behavior.

### 4.3 Evaluation of comprehensive fruit quality using the fruit quality index method

More information is needed on comprehensive quality assessments based on diversified quality indicators of different citrus fruits for optimizing selection. A thorough evaluation of fruit quality based on physical, chemical, biological, and other factors is an essential strategy for quantitatively assessing different fruit varieties and a critical method for improving high-quality fruit production and high-value-added deep processing (Neves et al., 2018; Klimek-Szczykutowicz et al., 2020; Khalil et al., 2022; Kumar et al., 2022). It has been widely reported that the IQI and NQI methods are effective ways to evaluate the comprehensive quality of a given thing with various indicators, including soil quality, water quality, and stress tolerance processes (Qi et al., 2009; Guo et al., 2019; Mukate et al., 2019; Guo et al., 2022; Sun et al., 2018). Therefore, it was also employed to assess the fruit quality in the present study. As a result, the lowest and highest FQI values were found in Maogugan and Ningmeng fruits among the investigated citrus cultivars for both the IQI and NQI methods (**Figure 7**), which implied that *Citrus limon* fruits have potential quality advantages for fresh consumption and product processing. Di Rauso Simeone et al. (2020) reported that lemon is the third most important citrus crop after orange and mandarin and is rich in numerous beneficial substances, including oils, antioxidants, and other phytochemicals. This finding was in line with a review by Klimek-Szczykutowicz et al. (2020) where the chemistry, pharmacological properties, applications in the modern pharmaceutical, food, and cosmetics industries, and biotechnological studies for lemon were summarized. Moreover, a positive relationship was observed between FQI_IQI_ and FQI_NQI_ across citrus fruits (**Figure 8**). These results indicated that the FQI method could be used to evaluate the various fruit quality indicators of citrus, and the quantitative value could be more intuitive to recommend fruit consumption or deep processing.

The PCA and dendrogram results consistently showed that there were four different quadrants and four main groups formed by the nine investigated citrus fruits from four different citrus types (**Figures 5**, **6**). Except for *Citrus limon* (including Ningmeng I and II fruits), these clusters also indicated that the investigated properties are independent of citrus types, implying that the fruit quality evaluation based on mineral elements and juice quality characteristics might be less affected by genetic and environmental conditions. Similar results were also observed by Guo et al. (2023), who noted that the differences among citrus fruits might be related not only to the nutritional features of the cultivar but also to the differences in climate, fertilization, soil type, and place of origin. In addition, according to the conception of the IQI and NQI methods, the IQI method calculates the sum of corresponding weight values of all the selected indicators and combined metrics into an index value by the SSF equation (Liebig et al., 2001; Guo et al., 2019; Guo et al., 2022); the NQI method mainly calculates the average and the minimum indicator score from the SSF equation for the selected indicators, but the indicator weights are not used (Qi et al., 2009). However, the value ranges of FQI_IQI_ and FQI_NQI_ were 0.382-0.590 and 0.106-0.245 in the present study, respectively, and the average value of FQI_IQI_ reached 3-fold higher than that of FQI_NQI_ (**Figure 7**). Further analysis showed that the mean values of peel, pulp, and seed mineral and juice quality properties contributing to the FQI_IQI_ were 25.99%, 28.10%, 29.11%, and 16.79% across the citrus fruits, which revealed that the seed mineral properties were responsible for a relatively higher contribution to the difference in the FQI (**Figure 7A**). These results suggest that the IQI method may be more conducive for quantifying the FQI than the NQI method, and the strategy for fruit quality assessment and fruit quality indicator selection should be further developed in the future.

## 5 Conclusions

To comprehensively evaluate fruit quality characteristics, this study first employed the IQI and NQI methods to assess the FQI by analysing the morphological, mineral, and juice properties of nine citrus fruit cultivars. There were significant differences in the investigated fruit quality indicators among the citrus fruits, which are rich in mineral elements in various fruit parts, further suggesting that citrus peel, pulp, and seed are valuable sources of macro- and micro-nutrients. Principal component analysis and dendrogram tests consistently demonstrated that the investigated citrus cultivars were clustered into four main groups, resulting in differences in the FQI, both based on the IQI and FQI methods. The mean value of FQI_IQI_ was three times higher than that of FQI_NQI_, and a positive correlation between FQI_IQI_ and FQI_NQI_ was identified, which indicated that the IQI and NQI methods were consistently evaluated for fruit quality assessment, with the IQI method being more precise. In conclusion, our findings reveal the differences in fruit quality characteristics among different citrus fruits and citrus fruit parts and provide a new strategy for fruit quality assessment, which can be widely used for the value-added utilization of fruits in citrus production and industry.

## Data availability statement

The original contributions presented in the study are included in the article/**Supplementary Material**. Further inquiries can be directed to the corresponding author.

## Author contributions

YLJ: Formal Analysis, Investigation, Visualization, Writing – original draft. SZ: Formal Analysis, Investigation, Visualization, Writing – original draft. HJ: Investigation, Methodology, Data curation, Visualization, Formal analysis, Writing – original draft. YW: Formal Analysis, Investigation, Methodology, Visualization, Writing – review & editing. YMJ: Formal Analysis, Investigation, Methodology, Visualization, Writing – review & editing. HZ: Formal Analysis, Investigation, Writing – review & editing. YYJ: Investigation, Formal analysis, Writing – review & editing. WL: Formal Analysis, Investigation, Writing – review & editing. L-SC: Resources, Supervision, Writing – review & editing. JG: Conceptualization, Data curation, Formal Analysis, Funding acquisition, Investigation, Methodology, Project administration, Resources, Software, Supervision, Validation, Visualization, Writing – original draft, Writing – review & editing.
