# Supplementary material for: Fruit quality assessment based on mineral elements and juice properties in nine citrus cultivars
Source: Front Plant Sci. 2023 Nov 30;14:1280495. doi: 10.3389/fpls.2023.1280495 (PMC10720443; doi:10.3389/fpls.2023.1280495)
Supplement: Supplementary file 1 [file DataSheet_1.docx]

**Supplementary information**

**Fruit quality assessment both mineral element and juice properties in 9 citrus cultivars**

Yiling Jiao^1†^, Shuozhen Zhang^1†^, Haitao Jin^1†^, Yuwen Wang^2^, Yamin Jia^1,3^, Hua Zhang^4^, Yuying Jiang^4^, Wenqiang Liao^4^, Li-Song Chen^1^, Jiuxin Guo^1*^

^1^ Fujian Provincial Key Laboratory of Soil Environmental Health and Regulation / International Magnesium Institute, College of Resources and Environment, Fujian Agriculture and Forestry University, Fuzhou 350002, China

^2^ Forestry Science and Technology Test Center of Fujian Province, Zhangzhou 363600, China

^3^ College of Forestry, Guangxi University, Nanning 530004, China

^4^ Station of Cropland Construction and Soil and Fertilizer of Fujian Province, Fuzhou 350003, China

**^†^** These authors have contributed equally to this work.

**^*^ Corresponding author:**

To whom correspondence should be addressed.

Name: Jiuxin Guo

E-mail: jiuxinguo@hotmail.com

Tel: ++86-591-83789361

Address: No.15 Shanxiadian Road, Cangshan District, Fuzhou City, Fujian Province, China 350002

**TABLE S1.** Results of PCA for all fruit indicators including peel, pulp, seed mineral characteristics and juice quality properties in different citrus cultivars.

| Principal component | PC1 | PC2 | PC3 | PC4 | PC5 | PC6 | PC7 |
| --- | --- | --- | --- | --- | --- | --- | --- |
| Eigenvalues | 12.596 | 6.932 | 4.568 | 3.570 | 2.803 | 2.613 | 1.501 |
| Percent (%) | 34.989 | 19.255 | 12.690 | 9.918 | 7.786 | 7.258 | 4.170 |
| Cumulative percent (%) | 34.989 | 54.244 | 66.934 | 76.852 | 84.638 | 91.896 | 96.065 |
| Eigenvectors/factors loading | | | | | | | |
| Peel N | -0.430 | 0.011 | -0.029 | 0.832 | 0.239 | -0.012 | -0.213 |
| Peel P | 0.477 | 0.171 | 0.388 | -0.208 | 0.087 | -0.433 | 0.298 |
| Peel K | 0.615 | 0.431 | -0.113 | 0.108 | 0.317 | -0.019 | -0.484 |
| Peel Ca | 0.861 | -0.368 | -0.304 | 0.051 | -0.149 | -0.021 | 0.035 |
| Peel Mg | -0.152 | 0.837 | -0.027 | 0.277 | -0.044 | -0.404 | -0.139 |
| Peel Fe | 0.738 | 0.085 | -0.510 | 0.127 | 0.020 | 0.282 | -0.296 |
| Peel Mn | 0.406 | 0.564 | 0.000 | 0.146 | 0.574 | 0.278 | -0.143 |
| Peel Cu | -0.064 | 0.245 | 0.757 | -0.451 | -0.206 | 0.274 | -0.187 |
| Peel Zn | -0.026 | 0.579 | 0.538 | -0.007 | -0.232 | 0.316 | 0.399 |
| Peel Al | -0.315 | 0.863 | -0.078 | -0.004 | 0.296 | 0.132 | 0.110 |
| Pulp N | 0.037 | 0.379 | 0.366 | 0.653 | -0.430 | -0.103 | -0.270 |
| Pulp P | 0.715 | 0.045 | 0.572 | 0.288 | 0.104 | -0.126 | 0.166 |
| Pulp K | 0.746 | 0.309 | 0.435 | -0.200 | 0.159 | -0.190 | -0.204 |
| Pulp Ca | 0.787 | -0.186 | -0.191 | 0.411 | -0.143 | -0.319 | 0.083 |
| Pulp Mg | -0.123 | 0.642 | 0.047 | 0.453 | 0.191 | -0.546 | 0.088 |
| Pulp Fe | 0.895 | -0.184 | -0.145 | 0.042 | 0.076 | 0.336 | -0.012 |
| Pulp Mn | 0.176 | -0.510 | 0.425 | 0.574 | -0.422 | -0.048 | 0.004 |
| Pulp Cu | 0.648 | -0.289 | 0.433 | 0.417 | 0.155 | -0.206 | 0.132 |
| Pulp Zn | 0.226 | -0.523 | 0.565 | 0.512 | 0.071 | 0.230 | 0.126 |
| Pulp Al | 0.899 | 0.278 | 0.032 | -0.059 | 0.190 | -0.163 | 0.202 |
| Seed N | 0.777 | 0.002 | -0.263 | 0.243 | -0.253 | 0.319 | 0.168 |
| Seed P | 0.753 | -0.160 | 0.307 | 0.077 | 0.154 | 0.474 | -0.055 |
| Seed K | -0.273 | 0.294 | -0.480 | 0.656 | -0.144 | 0.333 | 0.162 |
| Seed Ca | -0.391 | -0.695 | -0.092 | 0.120 | 0.454 | -0.333 | 0.060 |
| Seed Mg | -0.505 | 0.752 | 0.055 | 0.176 | -0.062 | 0.122 | 0.241 |
| Seed Fe | -0.298 | -0.431 | 0.222 | 0.003 | 0.714 | -0.174 | 0.036 |
| Seed Mn | -0.326 | 0.678 | -0.363 | 0.295 | 0.101 | 0.320 | 0.302 |
| Seed Cu | 0.659 | 0.453 | 0.423 | -0.254 | -0.122 | -0.261 | 0.108 |
| Seed Zn | 0.669 | 0.226 | 0.220 | 0.050 | 0.579 | 0.267 | -0.171 |
| Seed Al | -0.393 | -0.808 | 0.213 | 0.273 | -0.119 | 0.054 | -0.146 |
| Fruit TSS | -0.741 | -0.364 | -0.178 | -0.022 | 0.369 | 0.214 | 0.285 |
| Fruit TA | 0.939 | -0.159 | -0.204 | -0.064 | 0.014 | 0.200 | -0.030 |
| Fruit TSS/TA | -0.572 | 0.138 | 0.639 | -0.039 | -0.083 | 0.413 | -0.169 |
| Fruit pH | -0.820 | 0.129 | 0.454 | 0.042 | -0.122 | 0.101 | -0.271 |
| Fruit Vc | 0.906 | -0.087 | 0.071 | -0.074 | -0.084 | 0.199 | 0.267 |
| Fruit TP | -0.527 | -0.281 | 0.484 | 0.165 | 0.451 | 0.260 | 0.191 |

**TABLE S2.** Results of estimated communality and the weight value of each fruit quality indicator.

| Indicator | Communality | Weight |  | Indicator | Communality | Weight |
| --- | --- | --- | --- | --- | --- | --- |
| Peel N | 0.9804 | 0.0283 |  | Pulp Zn | 0.9798 | 0.0283 |
| Peel P | 0.7338 | 0.0212 |  | Pulp Al | 0.9924 | 0.0287 |
| Peel K | 0.9237 | 0.0267 |  | Seed N | 0.9251 | 0.0268 |
| Peel Ca | 0.9959 | 0.0288 |  | Seed P | 0.9448 | 0.0273 |
| Peel Mg | 0.9858 | 0.0285 |  | Seed K | 0.9803 | 0.0283 |
| Peel Fe | 0.9964 | 0.0288 |  | Seed Ca | 0.9803 | 0.0283 |
| Peel Mn | 0.9324 | 0.0270 |  | Seed Mg | 0.9323 | 0.0270 |
| Peel Cu | 0.9938 | 0.0287 |  | Seed Fe | 0.8657 | 0.0250 |
| Peel Zn | 0.9373 | 0.0271 |  | Seed Mn | 0.9886 | 0.0286 |
| Peel Al | 0.9681 | 0.0280 |  | Seed Cu | 0.9770 | 0.0282 |
| Pulp N | 0.9740 | 0.0282 |  | Seed Zn | 0.9849 | 0.0285 |
| Pulp P | 0.9774 | 0.0283 |  | Seed Al | 0.9651 | 0.0279 |
| Pulp K | 0.9844 | 0.0285 |  | Fruit TSS | 0.9766 | 0.0282 |
| Pulp Ca | 0.9875 | 0.0286 |  | Fruit TA | 0.9941 | 0.0287 |
| Pulp Mg | 0.9775 | 0.0283 |  | Fruit TSS/TA | 0.9620 | 0.0278 |
| Pulp Fe | 0.9773 | 0.0283 |  | Fruit pH | 0.9954 | 0.0288 |
| Pulp Mn | 0.9815 | 0.0284 |  | Fruit Vc | 0.9566 | 0.0277 |
| Pulp Cu | 0.9489 | 0.0274 |  | Fruit TP | 0.9265 | 0.0268 |


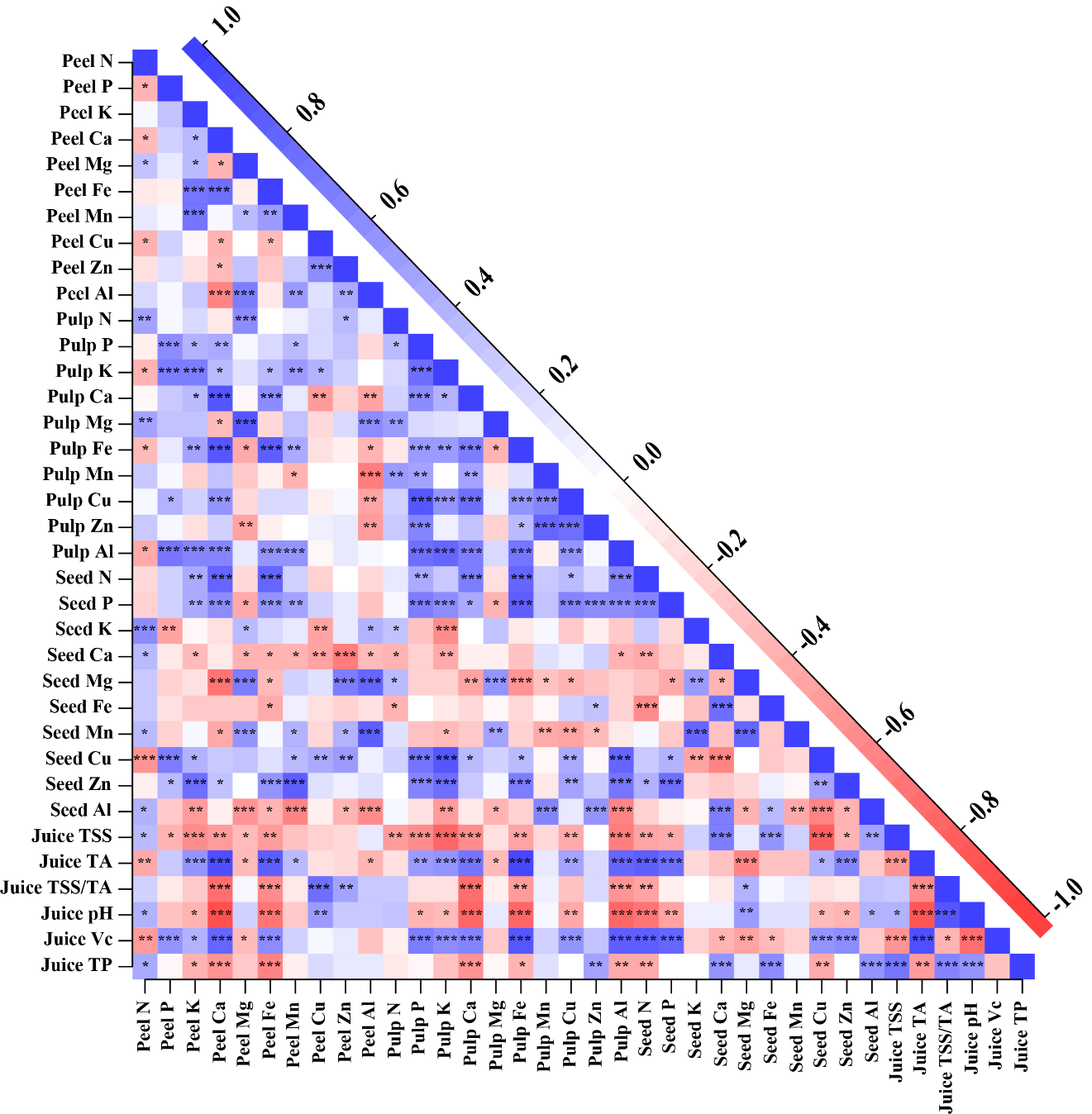


**FIGURE S1.** Correlation coefficients among peel, pulp, seed mineral characteristics and juice quality properties in different citrus cultivars. *, *p* < 0.05; **, *p* < 0.01; ***, *p* < 0.001.
